# Is Acceleration a Valid Proxy for Injury Risk in Minimal Damage Traffic Crashes? A Comparative Review of Volunteer, ADL and Real-World Studies

**DOI:** 10.3390/ijerph18062901

**Published:** 2021-03-12

**Authors:** Paul S. Nolet, Larry Nordhoff, Vicki L. Kristman, Arthur C. Croft, Maurice P. Zeegers, Michael D. Freeman

**Affiliations:** 1 CAPHRI School for Public Health and Primary Care, Faculty of Health, Medicine, and Life Sciences, Maastricht University, 6211 LM Maastricht, The Netherlands; p.nolet@maastrichtuniversity.nl (P.S.N.); m.zeegers@maastrichtuniversity.nl (M.P.Z.); 2 Private Practice, Pleasanton, CA 94565, USA; drnordhoff@comcast.net; 3 EPID@Work Research Institute, Department of Health Sciences, and the Division of Human Sciences, Northern Ontario School of Medicine, Lakehead University, Thunder Bay, ON P7B 5E1, Canada; vkristma@lakeheadu.ca; 4 Spine Research Institute of San Diego, San Diego, CA 92118, USA; arthur.croft59@gmail.com

**Keywords:** biomechanics, injury causation, rear impact crash, activities of daily living

## Abstract

Injury claims associated with minimal damage rear impact traffic crashes are often defended using a “biomechanical approach,” in which the occupant forces of the crash are compared to the forces of activities of daily living (ADLs), resulting in the conclusion that the risk of injury from the crash is the same as for ADLs. The purpose of the present investigation is to evaluate the scientific validity of the central operating premise of the biomechanical approach to injury causation; that occupant acceleration is a scientifically valid proxy for injury risk. Data were abstracted, pooled, and compared from three categories of published literature: (1) volunteer rear impact crash testing studies, (2) ADL studies, and (3) observational studies of real-world rear impacts. We compared the occupant accelerations of minimal or no damage (i.e., 3 to 11 kph speed change or “delta V”) rear impact crash tests to the accelerations described in 6 of the most commonly reported ADLs in the reviewed studies. As a final step, the injury risk observed in real world crashes was compared to the results of the pooled crash test and ADL analyses, controlling for delta V. The results of the analyses indicated that average peak linear and angular acceleration forces observed at the head during rear impact crash tests were typically at least several times greater than average forces observed during ADLs. In contrast, the injury risk of real-world minimal damage rear impact crashes was estimated to be at least 2000 times greater than for any ADL. The results of our analysis indicate that the principle underlying the biomechanical injury causation approach, that occupant acceleration is a proxy for injury risk, is scientifically invalid. The biomechanical approach to injury causation in minimal damage crashes invariably results in the vast underestimation of the actual risk of such crashes, and should be discontinued as it is a scientifically invalid practice.

## 1. Introduction

Traffic injury claims after minimal damage crashes make up a substantial portion of civil litigation. A major point of contention in such litigation is injury causation, with claimants most commonly relying on the opinion of a treating clinician to establish the crash as the cause of persisting injury [1]. Insurer defendants often rely on an engineering or “biomechanical approach” as a basis for denying the causal nexus between the crash and the claimed injuries [2,3]. The approach most commonly utilizes a sequential multi-step process [4]: first, the collision is reconstructed for severity, quantified by the speed change or “delta V” (ΔV) of the crash. Next, the reconstructed delta V is compared to experimental volunteer crash test studies, and the occupant accelerations from the studies (g forces, or “g’s”) are assigned to the investigated crash. Finally, the occupant forces attributed to the crash via the first two steps are compared to the forces described in studies of activities of daily living (ADLs), which allows for the conclusion that the risk of injury from the investigated crash was the same as the risk of injury from ADLs. This conclusion is presented to a judge or jury fact-finder to support the implication that the injury risk of the crash was essentially zero, based on the common experience that ADLs are benign events that virtually never cause injury. The biomechanical approach to causation allows the defendant to argue that a claimant’s clinically based determination of causation is not only incorrect, but also lacking in scientific rigor, as the clinician did not conduct a similar g force analysis demonstrating that the crash was indeed the cause of the diagnosed injuries.

By design, the defendant’s biomechanical causation approach will always result in the same conclusion; that a minimal damage crash was no more likely to cause a medically observed injury than forces encountered when walking or running, sitting down in a chair, head nodding, or by sneezing, etc. [5,6]. The conclusion is at odds with the results of large epidemiologic studies that indicate an injury rate of more than 20% in minimal and no damage crashes [7], and that such crashes result in several hundred thousand injuries diagnosed in U.S. emergency departments annually [8].

Further, the basic rationale underlying the first step of the biomechanical causation approach (reconstruction of the crash severity), which is that delta V is a reliable predictor of injury risk, has been called into question by the results of a recent longitudinal study of crash-related injuries, which reported that reconstructed crash severity is unrelated to the risk of developing chronic symptoms [9]. The findings are particularly relevant to crash injury litigation, given that it is the causation of chronic symptoms after a crash that are most likely to be disputed by an insurer, who in turn is more likely to employ a biomechanical expert to provide evidence to support the dispute [10].

The pivotal theory underlying the biomechanical causation approach, that the risk of injury in a minimal damage crash is the same as ADLs, cannot validly co-exist with the observational evidence that injuries from such crashes are relatively common, and that the most persisting injuries are unrelated to the severity of the crash. Thus, either the theory is false, or the observations reported across large populations are in error. If the theory is indeed false then it should be abandoned by medicolegal and engineering experts providing causation opinions in a legal setting, or, in the alternative, it should be uniformly labeled as unreliable and lacking in a valid scientific basis via the gatekeeper function of the courts, and thus inadmissible as evidence.

A fundamental operating premise of the biomechanical causation approach is that acceleration is a valid proxy for injury risk, and thus the evidence that the occupant acceleration of a crash is the same as the acceleration of an ADL translates to evidence that the risk of the injury from the two different events also the same.

This is a unique approach to estimating risk, and one that does not exist outside of the defense of injury litigation. Injury risk is defined as the probability that an injury will occur given a harmful exposure. Risk may be expressed as a per-exposure metric, or it may be adjusted to reflect a rate in a specified segment of the general population. As an example, the risk of an indoor fall in an elderly population has been reported as 328 per 1000 person-years, and the risk of a fracture from such a fall is 23 per 1000 person-years, based on a study of 980 Finnish subjects followed over 7 years [11]. Thus, the per-person risk of a fall in the study was 1 in 3, and the per-fall risk of a fracture was 1 in 14 (i.e., 23 per 328 falls). The risk of fall-related fracture was estimated using two critical pieces of information from the study population; the medical evidence of the injury, and the historically described circumstances in which the injury occurred. Additionally, the study population required sufficient numbers from which to infer statistically reliable estimates. The authors of the fall-related fracture study followed generally accepted epidemiologic methods for injury prevention, which begins with surveillance for occurrences of the injury [12].

In contrast with universally employed systematic approaches to the evaluation of injury risk, the biomechanical causation approach does not require, much less consider surveillance of injuries observed in populations of crash-exposed individuals. Indeed, the use of accelerations measured during ADLs as a proxy for injury risk not only allows for, but *requires* the circumvention of conventional epidemiologic methods for population-based risk assessment. Despite this bypassing of generally accepted scientific methods, published studies of ADL accelerations, with the express intent of providing an estimate of injury risk from traumatic events such as traffic crashes, have continued to accumulate over the past two decades. At the time of the preparation of this manuscript, there have been >30 publications describing the head accelerations of everyday activities, and more than a dozen of these have compared the results to the accelerations observed during experimental crash tests as a basis for assessing the risk of injury from real world crashes [13].

In the present study, the validity of the biomechanical approach to assessing minimal damage traffic crash injury causation is evaluated. The evaluation includes the following steps: (1) the literature describing volunteer crash tests conducted in the minimal to no damage range will be reviewed and summarized, allowing for a pooled analysis of occupant acceleration and other recorded metrics; (2) the literature describing ADL accelerations as a means of assessing crash-related injury risk will be reviewed and summarized, allowing for a pooled analysis of occupant acceleration; (3) epidemiologic studies of real world crashes occurring in the delta V range of volunteer crash tests will be reviewed and described; (4) all sources of data will be compared to assess the scientific validity of the use of acceleration as a proxy for injury risk.

## 2. Materials and Methods

### 2.1. Volunteer Crash Test Literature Review

The literature was searched for studies from the volunteer crash test literature that described rear impact crash tests of human volunteers. Studies included for analysis had the following characteristics: (1) they reported the delta V of the test (which was ≥3.2 km/h to exclude studies of “perturbations” rather than impacts); (2) included adults ≥18 years of age; and (3) provided information on individual crash tests, as opposed to aggregate reporting. Initially, studies were accessed from the personal libraries of the authors with prior publications regarding crash testing reviews and research publications on the topic (LN, AC, and MF) [14,15]. Next, PubMed and Google Scholar were searched for additional papers that described either rear impact crash testing of live human volunteers or reviews of such studies, and then the reference lists from the papers were examined for studies that were not discovered in the initial search. Proceedings of international car crash conferences were also searched for papers that were overlooked by the first 3 strategies, including the International Research Council on Biomechanics of Injury (IRCOBI), Society of Automotive Engineers (SAE), Association for the Advancement of Automotive Medicine (AAAM), and Enhanced Safety of Vehicles (ESV).

Studies were included for comprehensive review if they described either full scale crash testing (i.e., vehicle to vehicle) or sled testing that utilized a car seat, or bumper car impacts. The studies were initially identified as fulfilling the inclusion criteria by 3 authors (LN, AC, MF), then the data from the individual crash tests were abstracted and tabulated by two authors (LN, PN).

Data abstracted for each individually reported crash test included the following volunteer variables: age, gender, height, ethics approval/formal volunteer consent, state of preparedness for crash (braced/not braced). The presence of post-crash symptoms was coded as yes/no, with “yes” referring to any degree or duration of symptoms developed after the test. The highest single recorded or calculated peak head acceleration was determined for each test, regardless of vector (i.e., backwards or forwards, single or multiple [resultant]), or accelerometer arrangement (i.e., uniaxial vs. triaxial, located in a bite-block, head strap, or helmet) as well as for rearward and forward directions, reported in gravitational force equivalent (g force), with 1 g being equal to the force of the earth’s gravity (9.8 m/s^2^). Acceleration at T1 and L5 was also reported using g’s. The highest recorded angular head acceleration was determined for each test, and reported in rad/s^2^. Neck Injury Criterion (NIC), an index of injury risk based on the differential acceleration between the top and bottom of the cervical spine, was also recorded [16].

Abstracted information pertaining to the crash testing protocol included the type of impact apparatus (vehicle/sled/bumper car), the impact related speed change of the test vehicle (the delta V), which is defined as the maximum difference between pre and post impact speed in km/h, and the duration of the crash impulse in milliseconds (msecs).

### 2.2. Activities of Daily Living Literature

Studies that described linear and angular head acceleration recorded for various activities of daily living were identified from a previously published review paper by Miller et al. [13]. These authors identified the six most commonly described activities in the literature (i.e., ADLs described in more than a single paper), which consisted of walking, sitting, head nod, running, chair “plopping,” jumping down from a stair, and leaping. We abstracted the average and ranges of peak linear and angular head accelerations from the papers (95% confidence intervals could not be estimated from the grouped data), which were then put into a graphical format for comparison with the volunteer crash test data.

### 2.3. Observational Study of Injuries from Real-World Rear Impact Crashes

The final step of the literature review was a search for studies that provided individual subject information regarding injury from real world rear impact collisions that were reconstructed for speed change. Due to the high potential for large error rates in low-speed crash reconstruction methods [17], only papers that relied on downloaded airbag sensor (event data recorder [EDR]) information for estimating crash severity were included for review. Abstracted occupant information included presence, severity, and duration of injury. Abstracted information regarding the crash was derived from the downloaded EDR data, and included the average and peak vehicle acceleration in g’s, the duration of the crash pulse in msecs, and the delta V in km/h.

Abstracted data were put into an Excel file, and then transferred to either SPSS v26 (IBM SPSS Statistics for Windows, Version 26.0. IBM Corp., Armonk, NY, USA, or JMP 13 (JMP^®^, Version 13.0. SAS Institute Inc., Cary, NC, USA) for description, analysis, and graphing of crash test data and activities of daily living. Logistic regression modeling was used to examine the association in the crash testing data between delta V and peak body region acceleration or post-test symptoms, adjusting for test subject age and sex.

## 3. Results

### 3.1. Volunteer Crash Test Literature Review

**Figure 1:**
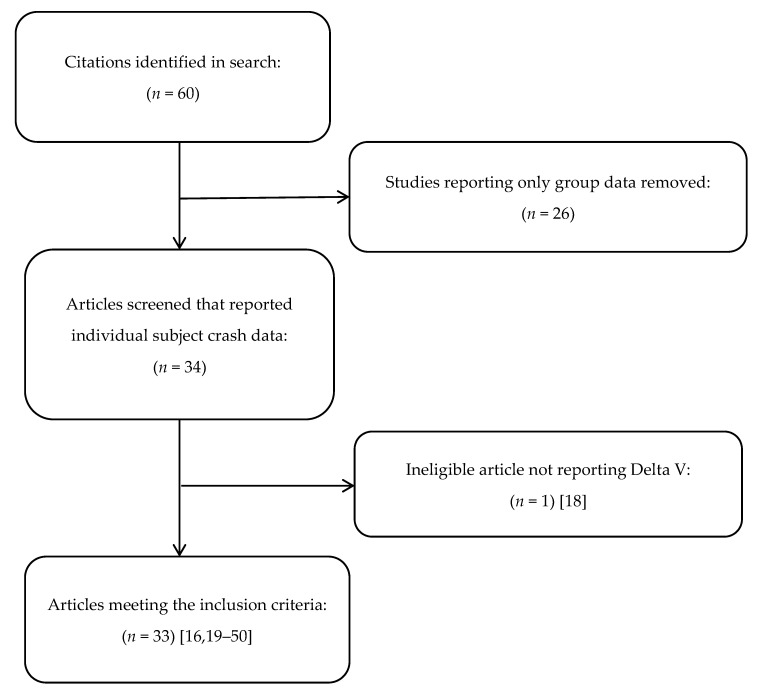
Identification and selection of articles describing rear-impact adult volunteer crash testing.

Our initial literature search resulted in the identification of 60 rear impact volunteer crash test studies, 26 of which were excluded as they reported only group crash data (See Figure 1). Of the remaining 34 studies, one was excluded as the authors reported barrier equivalent velocity (BEV) rather than delta V [18]. Among the 33 remaining studies there were data from 408 individual crash tests that could be abstracted for description and analysis [16,19,20,21,22,23,24,25,26,27,28,29,30,31,32,33,34,35,36,37,38,39,40,41,42,43,44,45,46,47,48,49,50]. The number of unique test subjects could not be determined from the studies, as multiple studies were performed by the same authors who also included themselves as test subjects, and most papers described two or more crash tests per subject (making the likely number of unique subjects in the included studies between 100 and 200). Approximately 5 out of 6 tests (83.3%) were performed on male subjects, and the average age, height, and weight of a test subject was 36.1 years, 174.9 cm, and 79.3 kg, respectively. There was no mention of ethical oversight and/or informed consent in approximately 1/3rd of the tests, a factor that also was significantly associated with higher average test delta V, which was 6.4 km/h across all tests. Other factors associated with higher test delta V were preparation for the impact, and the presence of symptoms after the test, which were reported after 37.5% of the crash tests (see Table 1).

**Figure 2:**
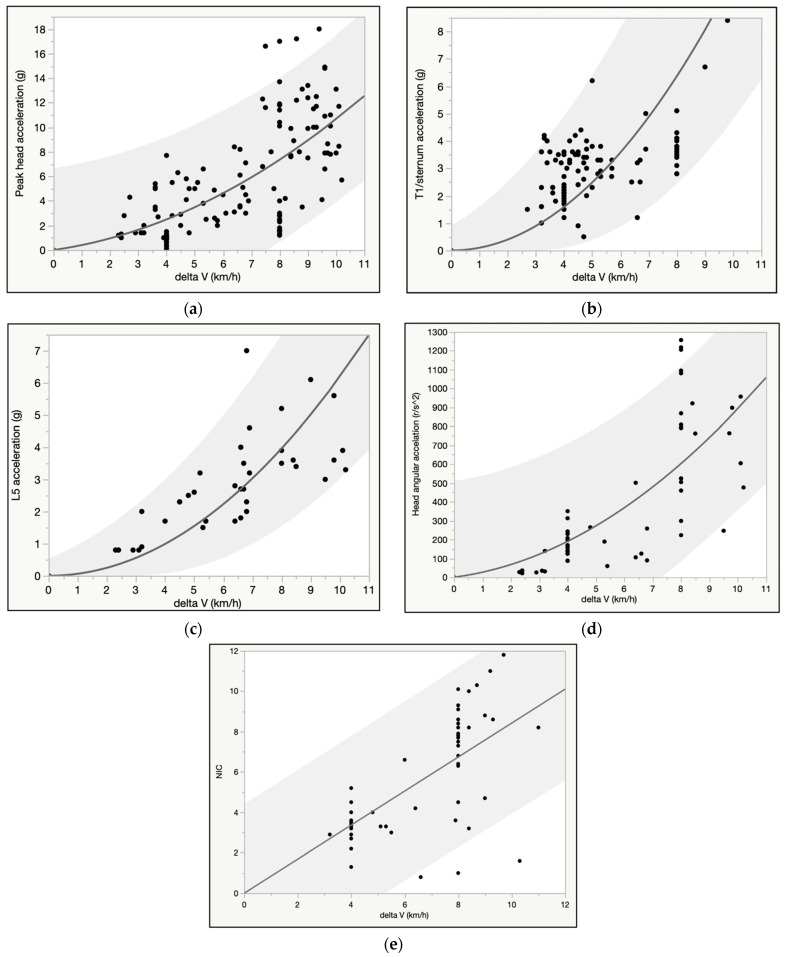
(**a**) (upper row left): Peak head acceleration (g) vs. delta V (km/h) for 126 crash tests. (**b**) (upper row right): Peak thoracic acceleration (g) vs. delta V (km/h) for 97 crash tests. (**c**) (middle row left): Peak L5 acceleration (g) vs. delta V (km/h) for 39 crash tests. (**d**) (middle row right): Angular head acceleration (rad/s^2^) vs. delta V (km/h) for 53 crash test subjects. (**e**) (lowest row): Neck Injury Criterion (NIC) vs. delta V (km/h) for 53 crash test subjects. T1: 1st thoracic vertebra. L5: 5th lumbar vertebra; NIC: Neck Injury Criterion. The dots in the charts indicate individual tests results, and the shaded area is the 95% predictive interval.

**Figure 3:**
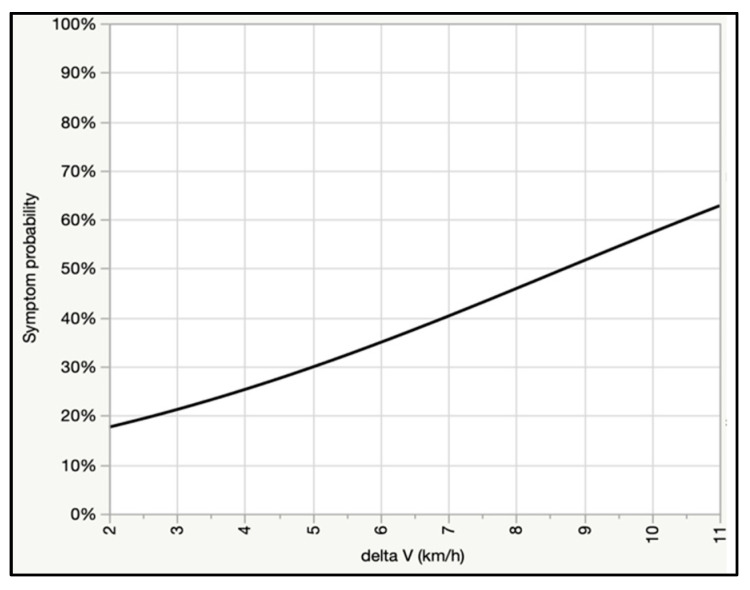
Risk of post-test symptoms for 381 crash tests.

We found a positive curvilinear association between delta V and peak head, T1, and L5 linear acceleration, as well as delta V and angular head acceleration, whereas the relationship between NIC and delta V was linear (Figure 2a–e). Peak head acceleration ranged broadly; as an example, at 8 km/h, average peak linear head acceleration was 7.4 g, but the lower and upper bound of the 95% predictive interval ranged from approximately 1 to 14 g, and the highest observed value was 17 g (Figure 2a). Angular head acceleration was likewise highly variable; at 8 km/h average angular acceleration was 605 rad/s^2^, but the lower and upper bound of the 95% predictive interval ranged from <100 to >1100 rad/s^2^, and the highest observed value was 1257 rad/s^2^ (Figure 2d). Risk of post-test symptoms was positively associated with delta V (Figure 3), and, in a multivariate logistic regression model, younger females were significantly more likely to report post-test symptoms than older male subjects at the same delta V. As an example, at the average delta V of the tests (6.4 km/h) there was a 71% probability of symptoms in a 20 year old female test subject versus a 34% risk in a 40 year old male test subject.

**Figure 4:**
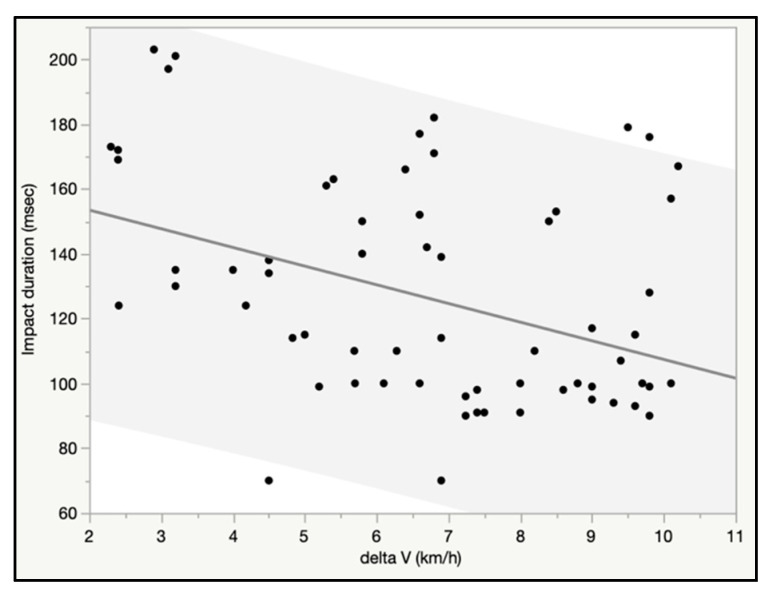
Impact duration (msec) vs. delta V (km/h) for 69 crash tests.

Average and median impact duration were 124 and 114 msec for 69 impacts, respectively. When regressed against delta V, there was a significant negative linear relationship for impact duration, and over 7 km/h the majority of impacts were less than 100 msecs (Figure 4).

### 3.2. Activities of Daily Living Literature

**Figure 5:**
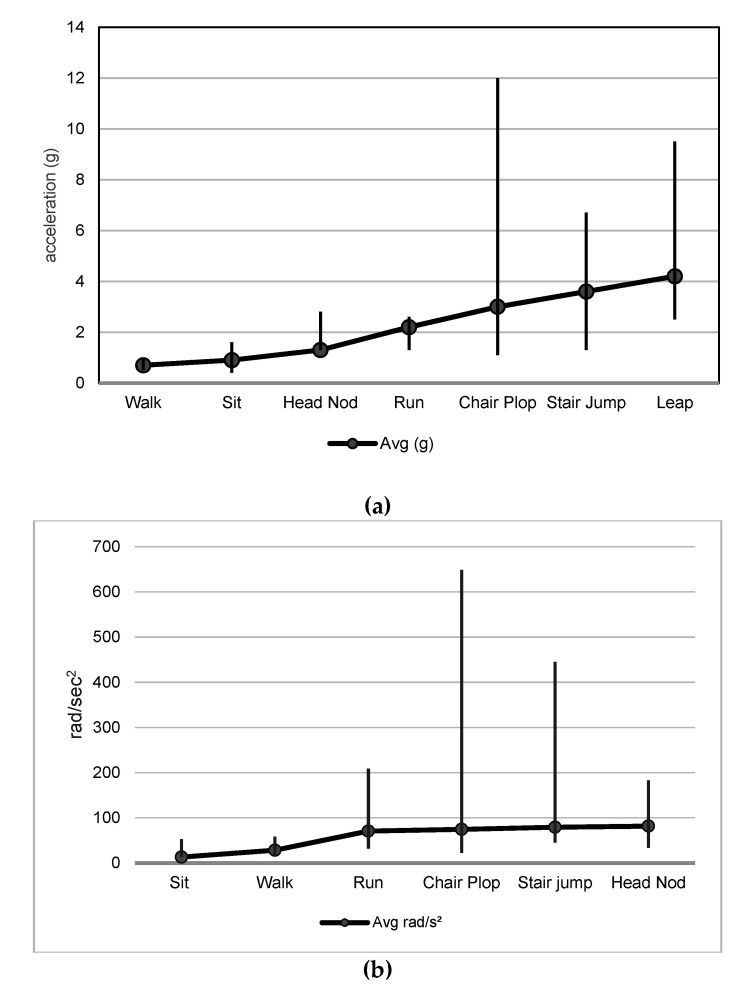
(**a**): Mean and range of peak linear head acceleration (g) measured for 6 ADLs. ADL: activities of daily living; Avg: average linear head acceleration in g. The vertical line illustrates the range of values for each ADL. (**b**) Mean and range of peak angular head acceleration (rad/s^2^) for 6 selected ADLs. ADL: activities of daily living; Avg: average peak angular acceleration in rad/s^2^. The vertical line illustrates the range of values for each ADL.

There were 7 and 4 studies describing linear and angular head accelerations, respectively, for the 6 ADLs selected for the pooled analysis [5,6,51,52,53,54,55,56]. Note: the studies were the same as those identified in the 2020 review paper by Miller et al. from which the 6 ADLs were selected [13]. A literature search revealed no additional relevant studies since the publication of this review. The mean peak linear head acceleration measurements (from 72 total subjects across 7 studies) progressively increased from walking and sitting (<1 g) to jumping from a stair and leaping (~4 g). Peak angular acceleration (from 58 subjects across 4 studies) was lowest, on average, for sitting in a chair (13.3 rad/s^2^) and highest for head nodding (81.7 rad/s^2^) (Table 2 and Figure 5a). While the “chair plop” activity averaged only 74.7 rad/s^2^, the activity was associated with the single highest outlier value of 649 rad/s^2^ (Table 3 and Figure 5b).

### 3.3. Observational Study of Injuries from Real-World Rear Impact Crashes

**Figure 6:**
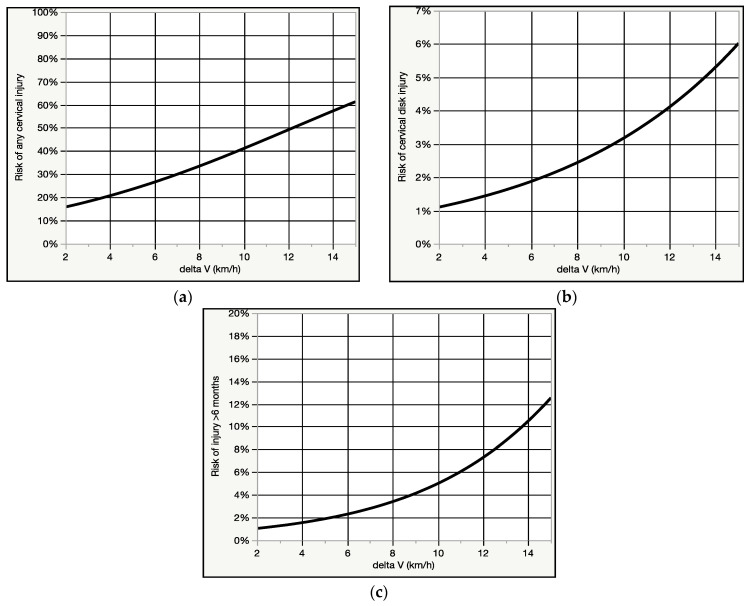
(**a**) Binomial fit of risk of any cervical spine injury vs. delta V (km/h) for 113 patients. (**b**) Binomial fit of risk of cervical disk/radiculopathy diagnosis vs. delta V (km/h) for 113 patients. (**c**) Binomial fit of risk of cervical spine injury lasting >6 months vs. delta V (km/h) for 114 patients.

Our literature search revealed 2 non-duplicative epidemiologic studies which described a total of 114 occupants and crashes that fit the inclusion criteria, allowing for the comparison of EDR-derived crash metrics to individual medical observations of injury presence, severity, and duration [57,58]. The injuries described in the studies were confined to the neck, and for the current analysis were variously dichotomized into (1) any neck injury (Grade I-III) versus no injury (Grade 0); (2) cervical injury with radicular/disk injury symptoms (i.e., Grade III) versus all other injury severities (Grade 0-II) [59], and (3) injury lasting more than 6 months, versus all other injury durations. Binomial regression was used to model risk curves of injury severity and duration over delta V (Figure 6a–c). Delta V was positively associated with injury presence, severity, and duration, as was mean and peak acceleration, however, sex was not a significant predictor of any of the injury outcomes examined.

**Figure 7:**
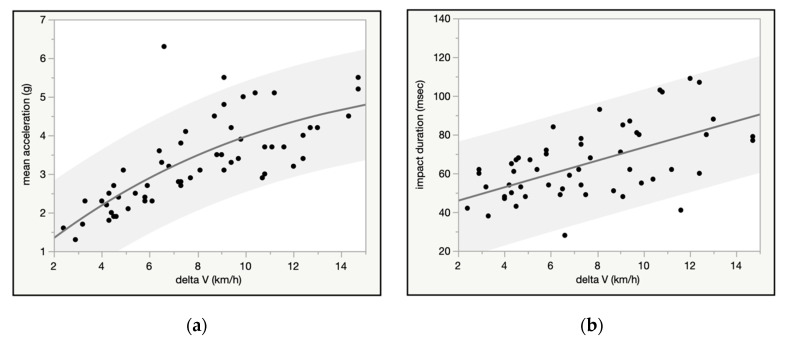
(**a**) Impact duration (msec) vs. delta V (km/h) for 94 real world crashes. (**b**) mean acceleration (g) vs. delta V (km/h) for 114 real world crashes.

The crash pulse (impact) duration and average and peak vehicle acceleration obtained from the EDR downloads were fit to the delta V. Average and median crash pulse duration was 75 and 68 msecs, respectively, and there was a positive linear association between impact duration and delta V, as well as with average and peak vehicle acceleration (Figure 7a,b). NB, although data from the 2 studies included crashes up to 32 km/h, the charts were truncated to 15 km/h to more closely match the data from the volunteer crash test literature.

### 3.4. Comparison of ADL Accelerations and Injury Risk to Rear Impact Accelerations and Injury Risk

Based on the results of our crash test data analysis, the highest mean linear peak head acceleration observed during an 11 km/h rear impact (12 g) is approximately 3 times the magnitude of the ADL with the highest average acceleration (i.e., leaping at 4 g), and the mean angular acceleration for an 11 km/rear impact (1150 rad/s^2^) is approximately 13 times greater than the average angular acceleration for any of the ADLs.

No literature was found that described the injury risk from ADLs, likely because ordinary ADLs do not cause injury in healthy people, and thus the topic has previously carried no research interest. An approximate upper boundary of risk can be estimated from common experience, however. It is reasonable to estimate the general population injury risk (requiring medical evaluation) from the ordinary ADLs described in the biomechanical literature and summarized above, such as walking or sitting down, at substantially less than 1 per year. This estimate is based on the common personal experience of the authors, and the generally true assumption that people do not routinely engage in ordinary activities that carry an appreciable risk of injury. Using the less than 1 per year occurrence assumption, we can perform an informal calculation of the injury risk for 2 of the activities that are described in the literature and used for comparison to crash testing; walking and sitting. The average person takes around 3000 steps per day, and sits down and gets up approximately 10–20 times per day. Thus, the annual risk of injury from these 2 activities would range from <1 per 1,095,000 steps to as much as <1 per 3650 acts of sitting (the denominator is calculated by multiplying the daily frequency of steps or sitting by 365 days in the year).

If we use the real world 11 km/h delta V rear impact injury risk from the present study (54%) and compare it to the highest estimated ADL-related risk (<1 in 3650 [0.027%] for sitting), then even using the most conservative estimates, the crash presents a risk of injury that is at least 2000 times greater than the “high risk” ADL of sitting. This ratio likely underestimates the actual injury risk disparity between rear impacts and ADLs by a factor of at least 10 times.

## 4. Discussion

Our analysis demonstrates that the theoretical basis for the biomechanical injury causation approach is scientifically invalid. As described in Section 3.4 above, the theory that serves as the operating principle for the methodology, that acceleration is a proxy for injury risk in low speed or minimal damage crashes, is demonstrably false. Even at the lowest levels of impact severity in a rear impact crash, the results of both crash testing and epidemiologic data from real-world crashes indicate a substantial (i.e., >20%) risk of at least some degree of injury. In contrast, everyday activities are benign events with virtually no injury risk whatsoever. If the magnitude of the accelerations resulting from crashes and ADLs can be said to be even roughly comparable, this fact only serves as further confirmatory evidence that occupant acceleration is not a proxy for injury risk.

There is no other example in the biomedical literature in which the established injury risk of any traumatic event is overlooked in favor of a comparison between the acceleration of the event and a non-injurious activity. Although there may be multiple shared attributes of traffic crashes and some ADLs, just as there are multiple shared attributes of stepping down from a stair and falling down a stair (i.e., the travel distances are the same, gravity is 9.81 m/s^2^ in both scenarios), alluding to the absence of injury while ordinarily walking down stairs sheds no light on the frequency of injury from falling down stairs. The comparison is inapt and should not be made.

Low speed or minimal damage rear impact crashes can be generally categorized as collisions with a delta V of <13 km/h (8 mph), as significant vehicle damage is unusual below this speed change in bumper to bumper impacts [20]. The risk of injury from such crashes has been described in the literature as ranging variously from a low of 12% (for <8 km/h [5 mph] delta V) to 47% for an 11 km/h (7 mph) delta V [60,61].

The real-world (epidemiologic) crash injury data analyzed for the current analysis, which provides the most reliable index of medically observed injury risk matched to delta V in the general population (as opposed to symptom risk in the population of crash test volunteers), indicated a 54% risk of any cervical spine injury, a 3.6% risk of a cervical disk injury/radiculopathy, and a 6% risk of an injury persisting for more than 6 months in an 11 km/h rear impact delta V crash [57,58]. While the observational studies used for the analysis are not comprehensive, as they only describe symptoms and diagnoses relating to the cervical spine, they do provide the most reliable indication of the general population injury response to low speed rear impact crashes at a specified delta V among the reviewed data.

These findings are consistent with the findings, by multiple authors, that no and minimal damage rear impact crashes are associated with a substantial risk of injury [9,62,63]. As an example, Chapline and colleagues reported no vehicle damage in 38% and 19% of collisions in which women and men were injured, respectively [64]. Bartsch et al. reported an average reconstructed delta V of 6.5 km/h (4 mph) among 113 study subjects who were injured in rear impact collisions, a speed at which no structural vehicle damage is typically observed in crash test studies [65]. Farmer et al. reported a 21% rate of injury in rear impact crashes with less than USD 500 in damage in a study involving injury claims made in 27 states in the US [7].

As we note in the present investigation, even crash testing among prepared, healthy, and largely young male volunteers is not an injury risk-free event; the risk of symptoms (persisting from hours to days) was previously reported as ranging from 28% to 63% for 5 to 12 km/h (3 to 7.5 mph) delta V tests, respectively [66]. Our updated analysis demonstrated similar symptom rates: 30% (95% CI 25%; 36%) and 68% (95% CI 55%; 89%) for 5 and 12 km/h.

The fact that a common-sense estimate demonstrates that the injury risk of a minimal to no damage rear impact crash is at least three orders of magnitude greater than the injury risk of any ADL has not stood as a barrier to the publication of a number of studies the purport to infer the injury risk of real-world traffic crashes from study of ADL accelerations. All of these studies are connected to the litigation defense community, either by author affiliation, financial support, or both.

The first such paper was published 1994, by Allen et al. [5]. The authors attached accelerometers to a bicycle helmet, and used the device to measure the head accelerations of 8 volunteers as they engaged in benign activities such as looking to the left, coughing, hopping off a step, and plopping into a chair. The authors used horizontal (x) and vertical (z) accelerations to calculate a resultant vector of acceleration. In comparing their results to motor vehicle crashes (MVC), the authors stated “Regarding day to day events, the present study analyzed the accelerations experienced at the head in volunteers examining the level of force that could be comparable to the under 8 km/h [5 mph], low velocity, no-damage rear-end motor vehicle incident,” and concluded that “the accelerations of the ADLs appeared to be *greater* than those seen in the same parts of the body during some no-damage motor vehicle rear-end impacts” (emphasis added). The study was financed by the Insurance Corporation of British Columbia (ICBC), for whom the first author provided expert testimony in court cases involving injury claims following low speed crashes [67].

In 2006, a study by Vijayakumar and colleagues compared the accelerations of volunteers exposed to low velocity (2.4 to 3.7 mph delta V) bumper car amusement park ride impacts to the accelerations measured on volunteers participating in various “vigorous” activities of daily living including hopping, skipping rope, falling into a chair, and running with an abrupt stop [68]. The peak resultant linear head accelerations reported for the 9 volunteers ranged from 2.8 to 9.9 g. The study was authored by employees of, and financed by a company that provides biomechanical causation analysis for insurer defendants in traffic crash injury litigation, Exponent, Inc.

Funk and colleagues from Biodynamic Research Corporation, another company that provides biomechanical causation analysis, published similar studies in 2007 and 2011, in which they compared rear impact collisions to what they claimed were “biomechanically similar” and “benign” daily activities [6,69]. The activities included head shaking, “plopping” down in a rigid seat, sustaining a vertical drop while seated (termed a “chair tip”), striking the forehead with the hand at 2.2 to 4.5 m/s, and an 8.5 and 11.5 m/s soccer ball impact to the head, *i.a*. The authors claimed that, “Several test scenarios in this study induced neck loading that was similar to a low speed (<10 km/h delta-V) [6.2 mph] automotive rear impact in both magnitude and direction.”

In a 2010 conference paper financed by Exponent Inc., Bussone and Duma described head angular acceleration of 18 volunteers engaging in a variety of daily activities, including plopping into a chair, head bobbing, looking quickly to the left, walking at a speed of 1.3 m/s, running at a speed of 2.7 m/s, performing maximum vertical leaps, and jumping off of a 20 cm high step, among others. The stated purpose of the study was to establish the “upper level of known non-injurious loading” at which a concussion would not occur, concluding that a “healthy adult” can be reasonably expected to sustain up to 931 rad/s^2^ of angular acceleration without risk of injury [56].

Most recently (2018), Cormier and colleagues (all employees of Biodynamic Research Corporation), described a review of crash testing literature and an analysis of national crash injury data pertaining to rear impact crashes, noting that the latter indicated a 12% and 22% rate of cervical spine injury at <8 km/h and 8–16 km/h, respectively [60]. Despite this finding, the authors discussed the similarity between the forces of ADLs and the crashes, and concluded that an 18 km/h rear impact crash test is a safe event. These results were later described as “meaningless” in a subsequent study, as the cited national crash injury database analysis consisted of a sample of an average of 1.3 crashes occurring at <8 km/h *per year* in the United States, which represented fewer than 1 in 100,000 of the annual injuries from similar crashes [8]. Even if the 12% to 22% injury rate were accurate, however, an event that results in a visit to a hospital emergency department in as many as 1 in 5 occurrences cannot be logically compared to any non-injurious activity of daily living.

It is worthwhile noting that while no significant (i.e., spinal disk or fracture) injuries were reported in the reviewed volunteer crash test studies, the rate of symptoms after testing is remarkably high. It is well established that experimental crash tests, as well as the volunteers who agree to be subjected to them, are sufficiently dissimilar to the circumstances and occupants of real-world crashes such that the injury risk of the latter cannot be inferred from the former [70,71]. While it is reasonable that crash testing of volunteers can be conducted in a manner that minimizes injury risk, and that at relatively low crash severities (i.e., <8 km/h delta V) the risk of significant injury to a prepared and healthy volunteer is quite low, it is also notable that the risk of chronic (i.e., >6 months) and significant (i.e., spinal disk/radiculopathy) injury at the same delta V in a real world crash is 3.3% (1 in 30) and 2.4% (1 in 42), respectively, based on the results of the current investigation. It is reasonable to postulate that it is only matter of time before a volunteer is significantly injured in a rear impact crash test, and this very real possibility should be a factor in any Institutional Review Board assessment of a proposed crash test study involving live human volunteers.

## 5. Conclusions

Our results indicate that there is enormous disparity between the risk of injury from ADLs and minimal damage crashes, and that the former cannot be used as a proxy for the latter. While the upper bound of the peak head accelerations observed for *some* ADLs overlap with the lower bound of peak head accelerations observed in *some* no damage rear impact crash tests, the risk of injury from most minimal damage crashes (at least 20%) is thousands of times greater than for any ADL. These results demonstrate that the biomechanical approach utilized in the legal defense of injury claims, in which the injury risk of a minimal damage rear impact crash is deemed comparable to the injury risk of ADLs, is an invalid method for evaluating injury causation. The approach should be discontinued, or in the alternative, ruled inadmissible by courts as its use results in unreliable expert testimony.

## Figures and Tables

**Table 1:** Subject demographics and crash test characteristics, vs. test delta V.

Crash Test Variable	*n* (%)	Mean Delta V, (95% CI) [km/h]
Gender, total	407	*p* = 0.70
Female	68 (16.7)	6.3 (5.7–6.9)
Male	339 (83.3)	6.8 (6.3–7.2)
Ethics/Consent, total	380	*p* < 0.001
Yes	248 (65.3)	6.2 (5.7–6.6)
No	132 (34.7)	6.6 (6.3–6.9)
Subject state, total	298	*p* = 0.002
Prepared	108 (36.2)	7.1 (6.7–7.4)
Unprepared	190 (63.8)	6.5 (6.1–6.9)
Post-test symptoms reported, total	384	*p* < 0.001
Yes	144 (37.5)	7.2 (6.7–7.6)
No	240 (62.5)	6.0 (5.7–6.3)
Vehicle Type Total	407	*p* < 0.001
Vehicle	272 (66.8)	6.8 (6.5–7.1)
Sled	127 (31.2)	5.5 (5.3–5.8)
Bumper Car	8 (2.0)	7.0 (6.5–7.6)
**Crash Test Subjects**	***n***	**Mean (SD)**
Age (years)	331	36.1 (10.7)
Height (cm)	275	174.9 (9.9)
Weight (Kg)	275	79.3 (17.6)
Delta V (km/h)	408	6.4 (2.3)
Highest measured acceleration of head (g)	129	5.9 (4.5)
Acceleration at head forward (g)	80	4.1 (3.8)
Acceleration at head rearward (g)	66	−2.2 (1.4)
Acceleration at sternum/T1 (g)	97	3.1 (1.2)
Acceleration at L5 (g)	39	2.9 (1.5)
Head angular acceleration (rad/s^2^)	53	−413.9 (369.2)
Impact duration (msec)	69	125.6 (33.7)
NIC Max	51	5.6 (2.9)

NIC: Neck Injury Criterion; T1: 1st thoracic vertebra.

**Table 2:** Activities of daily living (ADL), highest head acceleration (g).

ADL (g)		Allen 1994	Bussone 2005	Carriot 2014	Funk 2011	Kavanagh 2004	Kuo 2017	Ng 2006	Pooled Avg (g)	Total Subjects (*n*)
Walk	Subjects (*n*)		18	8		16		18		60
	Avg (g)		0.7	1.0		0.5		0.7	0.7	
	High (g)		1.0					1.0	1.0	
	Low (g)							0.5	0.5	
Sit Down	Subjects (*n*)		18					18		36
	Avg (g)		0.8					0.9	0.9	
	High (g)		0.8					1.4	1.6	
	Low (g)							0.4	0.4	
Head Nod	Subjects (*n*)		18							18
	Avg (g)		1.3						1.3	
	High (g)		2.8						2.8	
	Low (g)									
Run	Subjects (*n*)		18	8			2	18		46
	Avg (g)		1.6	4.1			3.6	1.7	2.2	
	High (g)		2.6					2.6	2.6	
	Low (g)							1.3	1.3	
Chair Plop	Subjects (*n*)	8	18		20			18		64
	Avg (g)	4.4	2.0		3.7			2.4	3.0	
	High (g)	8.5	4.0		12.0			4.0	12.0	
	Low (g)	1.8			2.5			1.1	1.1	
Hop off Stair	Subjects (*n*)	8	18		20			18		64
	Avg (g)	4.6	3.1		3.9			3.3	3.6	
	High (g)	6.7	4.2		6.1			5.0	6.7	
	Low (g)	1.3			1.7			2.0	1.3	
Vertical Leap	Subjects (*n*)			8				18		26
	Avg (g)			2.9				4.7	4.2	
	High (g)							9.5	9.5	
	Low (g)							2.5	2.5	

**Table 3:** Activities of Daily Living, angular head acceleration (rad/s^2^).

ADL		Bussone 2005	Bussone 2010	Funk 2011	Kuo 2017	Pooled Avg (rad/s^2^)	Total Subjects (*n*)
Sit Down	Subjects (*n*)	18					18
	Avg (rad/s^2^)	13.3				13.3	
	High (rad/s^2^)	52.6				52.6	
	Low (rad/s^2^)						
Walk	Subjects (*n*)	18	18				36
	Avg (rad/s^2^)	24.9	31.9			28.4	
	High (rad/s^2^)	48.6	58.0			58.0	
	Low (rad/s^2^)		13.8			13.8	
Run	Subjects (*n*)	18	18		2		38
	Avg (rad/s^2^)	66.1	80.1		161.0	70.7	
	High (rad/s^2^)	208.7	208.7			208.7	
	Low (rad/s^2^)		31.6			31.6	
Head Nod	Subjects (*n*)	18	18				36
	Avg (rad/s^2^)	73.8	89.7			81.7	
	High (rad/s^2^)	73.9	183.1			183.1	
	Low (rad/s^2^)		33.9			33.9	
Chair Plop	Subjects (*n*)	18	18	20			56
	Avg (rad/s^2^)	84.5	129.1	169.0		74.7	
	High (rad/s^2^)	320.3	320.2	649.0		649.0	
	Low (rad/s^2^)		29.2	22.0		22.0	
Hop off Stair	Subjects (*n*)	18	18	20			56
	Avg (rad/s^2^)	85.6	206.4	68.0		79.3	
	High (rad/s^2^)	232.6	445.1	170.0		445.1	
	Low (rad/s^2^)		44.8	27.0		44.8	

Avg: average angular head acceleration; high: highest angular head acceleration; low: lowest angular head acceleration.

## Data Availability

Not applicable.

## References

[1] Freeman M.D., Centeno C.J., Kohles S.S. (2009). A Systematic Approach to Clinical Determinations of Causation in Symptomatic Spinal Disk Injury Following Motor Vehicle Crash Trauma. PM&R.

[2] Hayes W.C., Erickson M.S., Power E.D. (2007). Forensic injury biomechanics. Annu. Rev. Biomed. Eng..

[3] Walz F.H., Muser M.H. (2000). Biomechanical assessment of soft tissue cervical spine disorders and expert opinion in low speed collisions. Accid. Anal. Prev..

[4] Ogden J.S. (1999). Forensic Engineering Analysis of Damage and Restitution in Low Velocity Impacts. J. Natl. Acad. Forensic Eng..

[5] Allen M.E., Weir-Jones I., Motiuk D.R., Flewin K.R., Goring R.D., Kobetitch R. (1994). Acceleration perturbations of daily living a comparison to whiplash. Spine.

[6] Funk J.R., Cormier J.M., Bain C.E., Guzman H., Bunugi E. (2011). Head and Neck Loading in Everyday and Vigorous Activities. Ann. Biomed. Eng..

[7] Farmer C.M., Wells J.K., Werner J.V. (1999). Relationship of head restraint positioning to driver neck injury in rear-end crashes. Accid. Anal. Prev..

[8] Freeman M.D., Leith W.M. (2020). Estimating the number of traffic crash-related cervical spine injuries in the United States; An analysis and comparison of national crash and hospital data. Accid. Anal. Prev..

[9] Elliott J.M., Heinrichs B.E., Walton D.M., Parrish T.B., Courtney D.M., Smith A.C. (2019). Motor vehicle crash reconstruction: Does it relate to the heterogeneity of whiplash recovery?. PLoS ONE.

[10] Centeno C.J., Freeman M.D. (2005). A review of the literature refuting the concept of minor impact soft tissue injury. Pain Res. Manag..

[11] Luukinen H., Herala M., Koski K., Honkanen R., Laippala P., Kivelä S.-L. (2000). Fracture Risk Associated with a Fall According to Type of Fall Among the Elderly. Osteoporos. Int..

[12] Center for Injury Prevention and Control (2020). Our Approach Injury Center Centers for Disease Control, Our Approach. https://www.cdc.gov/injury/about/approach.html.

[13] Miller L.E., Urban J.E., Whelan V.M., Baxter W.W., Tatter S.B., Stitzel J.D. (2020). An envelope of linear and rotational head motion during everyday activities. Biomech. Model. Mechanobiol..

[14] Croft A.C., Haneline M.T., Freeman M.D. (2002). Low speed frontal crashes and low speed rear crashes: Is there a differential risk for injury?. Annu. Proc. Assoc. Adv. Automot. Med..

[15] Nordhoff L., Freeman M.D., Siegmund G.P. (2007). Human Subject Crash Testing: Innovations and Advances.

[16] Croft A.C., Herring P., Freeman M.D., Haneline M.T. (2002). The neck injury criterion: Future considerations. Accid. Anal. Prev..

[17] Bartlett W., Wright M., Masory O., Brach R., Baxter A., Schmidt B., Navin F. (2002). Evaluating the Uncertainty in Various Measurement Tasks Common to Accident Reconstruction. SAE Int..

[18] West D.H., Gough J.P., Harper G.T.K. (1993). Low Speed Rear-End Collision Testing Using Human Subjects. Accid. Reconstr. J..

[19] Anderson R.D., Welcher J.B., Szabo T.J., Eubanks J.J. (1998). Effect of Braking on Human Occupant and Vehicle Kinematics in Low Speed Rear-End Collisions. Soc. Automot. Eng..

[20] Bailey M.N., Wong B.C., Lawrence J.M. (1995). Data and Methods for Estimating the Severity of Minor Impacts. SAE Trans..

[21] Braun T.A., Jhoun J.H., Braun M.J., Wong B.M., Boster T.A., Kobayashi T.M., Perez F.A. (2001). Rear-End Impact Testing with Human Test Subjects. Soc. Automot. Eng..

[22] Castro W.H.M., Schilgen M., Meyer S., Weber M., Peuker C., Wörtler K. (1997). Do ‘whiplash injuries’ occur in low-speed rear impacts. Eur. Spine J..

[23] Croft A.C., Philippens M.M.G.M. (2006). The RID2 Biofidelic Rear Impact Dummy: A Validation Study Using Human Subjects in Low Speed Rear Impact Full Scale Crash Tests: Neck Injury Criterion (NIC). No. 2006-01-0067.

[24] Davidsson J., Deutscher C., Hell W., Linder A. Human Volunteer Motion in Rear-End Impacts. Proceedings of the 1998 International IRCOBI Conference on the Biomechanics of Impact.

[25] Erikson M.S. (2012). Fidelity of Biodynamic Simulation Models for Low Speed Collinear Rear Crash Conditions. SAE Technical Paper No. 2012-01–0570.

[26] Fugger T.F., Randles B.C., Welcher J.B., Szabo T.J. (2003). Vehicle and Occupant Kinematics in Low-Speed Override/Underride Collisions: SAE Technical Paper No. 2003-01–0158.

[27] Furbish C., Ivory M., Hoffman M., Anderson R., Anderson R. (2011). Steering Column Loads and Upper Extremity Motions During Low Speed Rear-End Collisions: SAE Technical Paper No. 2011-01–0275.

[28] Goodwin V., Martin D., Sackett R., Schaefer G., Olson D., Tencer A. (1999). Vehicle and Occupant Response in Low peed Car to Barrier Override Impacts: SAE Technical Paper No. 1999-01–0442.

[29] Henderson B.J. (2012). A Retrospective Study in Understanding ‘Low Speed Change Vehicle Collisions, Occupant Movement’ and Likelihood of Injury. Ph.D. Thesis.

[30] Hong S.W., Park S.J., Lee Y.N., Yoo J.H., Kim H. (2013). Low-speed rear impact sled tests involving human subjects. Ann. Adv. Automot. Med..

[31] Hoyes P., Henderson B. (2013). A study and comparison of the effects of low speed change vehicle collisions on the human body. Accid. Anal. Prev..

[32] Ivory M.A., Furbish C.J., Hoffman M.R., Miller E.R., Anderson R.L., Anderson R.D. (2010). Brake Pedal Response and Occupant Kinematics During Low Speed Rear-End Collisions: SAE Technical Paper No. 2010-01–0067.

[33] Linder H., Lovsund A., Steffan P. (1999). Validation of the BioRID P3 Against Volunteer and PMHS Test Data and Comparison to the Hybrid III in Low-Velocity Rear-End Impacts. Annu. Proc. Assoc. Adv. Automot. Med..

[34] Matsushita T., Sato T.B., Hirabayashi K., Fujimura S., Asazuma T., Takatori T. (1994). X-ray Study of the Human Neck Motion due to Head Inertia Loading: SAE Technical Paper No. 942208.

[35] McConnell W.E., Howard R.P., Guzman H.M., Bomar J.B., Raddin J.H., Benedict J.V., Smith H.L. (1993). Analysis of Human Test Subject Kinematic Responses to Low Velocity Rear End Impacts: SAE Technical Paper No. 930889.

[36] McConnell W.E., Howard R.P., Poppel J.V., Krause R., Guzman H.M., Bomar J.B., Raddin J.H., Benedict J.V. (1995). Human Head and Neck Kinematics after Low Velocity Rear-End Impacts-Understanding Whiplash: SAE Technical Paper Nr. 952724.

[37] McConnell W.E., Guzman H.M., Krenrich S.W., Bomar J.B., Harding R.M., Raddin J.H., Funk J.R. (2003). Human kinematics during non-collinear low velocity rear end collisions. Annu. Proc. Assoc. Adv. Automot. Med..

[38] Ono K., Kaneoka K., Inami S. Influence of seat properties on human cervical vertebral motion and head/neck/torso kinematics during rear-end impacts. Proceedings of the International IRCOBI Conference on the Biomechanics of Impact.

[39] Ono K., Ejima S., Suzuki Y., Kaneoka K., Fukushima M., Ujihashi S. Prediction of Neck Injury Risk Based on the Analysis of Localized Cervical Vertebral Motion of Human Volunteers During Low-Speed Rear Impacts. Proceedings of the International IRCOBI Conference on the Biomechanics of Impact.

[40] Rosenbluth W., Hicks L. (1994). Evaluating Low-Speed Rear-End Impact Severity and Resultant Occupant Stress Parameters. J. Forensic Sci..

[41] Sato F., Nakajima T., Ono K., Svensson M., Brolin K., Kaneoka K. Dynamic Cervical Vertebral Motion of Female and Male Volunteers and Analysis of its Interaction with Head/Neck/Torso Behavior during Low-Speed Rear Impact. https://publications.lib.chalmers.se/records/fulltext/207480/local_207480.pdf.

[42] Schmidt B.F., Haight W.R., Szabo T.J., Welcher J.B. (1998). System-Based Energy and Momentum Analysis of Collisions: SAE Technical Paper Nr. 980026.

[43] Scott M.W., McConnell W.E., Guzman H.M., Howard R.P., Bomar J.B., Smith H.L., Benedict J.V., Raddin J.H., Hatsell C.P. (1993). Comparison of Human and ATD Head Kinematics During Low-Speed Rearend Impacts: SAE Technical Paper Nr. 930094.

[44] Siegmund G.P. Speed change (ΔV) of amusement park bumper cars. Proceedings of the Canadian Multidisciplinary Road Safety Conference VIII.

[45] Szabo T.J., Welcher J.B., Anderson R.D., Rice M.M. (1994). Human Occupant Kinematic Response to Low Speed Rear-End Impacts: SAE Technical Paper No. 940532.

[46] Szabo T.J., Welcher J.B. (1996). Human Subject Kinematics and Electromyographic Activity during Low Speed Rear Impacts: SAE Technical Paper Nr. 962432.

[47] Tanner C.B., Chen H.F., Wiechel J.F., Brown D.R. (1997). Vehicle and Occupant Response in Heavy Truck to Car Low-Speed Rear Impacts: SAE Technical Paper Nr. 970120.

[48] Watanabe S., Ichikawa H., Kayama O., Ono K., Kaneoka K., Inami S. (1999). Relationships Between Occupant Motion and Seat Characteristics in Low-Speed Rear Impacts: SAE Technical Paper Nr. 1999-01–0635.

[49] Welcher J.B., Szabo T.J. (2001). Relationships Between Seat Properties and Human Subject Kinematics in Rear Impact Tests. Accid. Anal. Prev..

[50] Welcher J.B., Szabo T.J. (2001). Human Occupant Motion in Rear-End Impacts: Effects of Incremental Increases in Velocity Change: SAE Technical Paper Nr. 2001-01–8999.

[51] Kavanagh J.J., Barrett R.S., Morrison S. (2004). Upper body accelerations during walking in healthy young and elderly men. Gait Posture.

[52] Bussone W.R. (2005). Linear and Angular Head Accelerations in Daily Life.

[53] Ng T.P., Bussone W.R. (2006). The effect of gender and body size on linear accelerations of the head observed during daily activities. Biomed. Sci. Instrum..

[54] Carriot J., Jamali M., Chacron M.J., Cullen K.E. (2014). Statistics of the vestibular input experienced during natural self-motion: Implications for neural processing. J. Neurosci..

[55] Kuo C., Wu L.C., Yu P.P., Laksari K., Camarillo D.B., Kuhl I. (2017). Pilot findings of brain displacements and deformations during roller coaster rides. J. Neurotrauma.

[56] Bussone W.R., Duma S.M. (2010). The effect of gender and body size on angular accelerations of the head observed during everyday activities. Biomed. Sci. Instrum..

[57] Krafft M., Kullgren A., Ydenius A., Tingvall C. (2002). Influence of Crash Pulse Characteristics on Whiplash Associated Disorders in Rear Impacts--Crash Recording in Real Life Crashes. Traffic Inj. Prev..

[58] Ono K., Ejima S., Yamazaki K., Sato F., Pramudita J.A., Kaneoka K., Ujihashi S. Evaluation criteria for the reduction of minor neck injuries during rear-end impacts based on human volunteer experiments and accident reconstruction using human FE model simulations. Proceedings of the International Research Council on the Biomechanics of Impact (IRCOBI) Conference.

[59] Sterling M., Sterling M., Kenardy J. (2011). Whiplash: Evidence Base for Clinical Practice.

[60] Cormier J., Gwin L., Reinhart L., Wood R., Bain C. (2018). A Comprehensive Review of Low-Speed Rear Impact Volunteer Studies and a Comparison to Real-World Outcomes. Spine.

[61] Freeman M.D. Biomechanical, Mechanical, and Epidemiologic Characteristics of Low Speed Rear Impact Collisions. Proceedings of the 67th Annual Meeting of the American Academy of Forensic Sciences.

[62] Croft A.C., Freeman M.D. (2005). Correlating crash severity with injury risk, injury severity, and long-term symptoms in low velocity motor vehicle collisions. Med. Sci. Monit..

[63] Robbins M.C. (1997). Lack of Relationship between Vehicle Damage and Occupant Injury: SAE Technical Paper Nr. 970494.

[64] Chapline J.F., Ferguson S.A., Lillis R.P., Lund A.K., Williams A.F. (2000). Neck pain and head restraint position relative to the driver’s head in rear-end collisions. Accid. Anal. Prev..

[65] Bartsch A.J., Gilbertson L.G., Prakash V., Morr D.R., Wiechel J.F. (2008). Minor crashes and ‘whiplash’in the United States. Ann. Adv. Automot. Med. Sci. Conf..

[66] Moss R.T., Bardas A.M., Hughes M.C., Happer A.J. (2005). Injury Symptom Risk Curves for Occupants Involved in Rear End Low Speed Motor Vehicle Collisions: SAE Technical Paper Nr. 2005-01-0296.

[67] (1991). La Flamme v. Stevens.

[68] Vijayakumar V., Scher I., Gloeckner D.C., Pierce J., Bove R., Young D. (2006). Head Kinematics and Upper Neck Loading during Simulated Low-Speed Rear-End Collisions: A Comparison with Vigorous Activities of Daily Living: SAE Technical Paper Nr. 0247.

[69] Funk J.R., Cormier J.M., Bain C.E., Guzman H., Bonugli E. An Evaluation of Various Neck Injury Criteria in Vigorous Activities. https://brconline.com/publications/an-evaluation-of-various-neck-injury-criteria-in-vigorous-activities/.

[70] Society of Automotive Engineers International (2011). SAE J885: Human Tolerance to Impact Conditions as Related to Motor Vehicle Design.

[71] Freeman M.D., Croft A.C., Rossignol A.M., Weaver D.S., Reiser M. (1999). A review and methodologic critique of the literature refuting whiplash syndrome. Spine.

